# The effect of *Irvingia gabonensis *seeds on body weight and blood lipids of obese subjects in Cameroon

**DOI:** 10.1186/1476-511X-4-12

**Published:** 2005-05-25

**Authors:** Judith L Ngondi, Julius E Oben, Samuel R Minka

**Affiliations:** 1 Nutrition, HIV and Health Research Unit, Department of Biochemistry, P.O Box 812, Faculty of Science, University of Yaounde I, Cameroon

## Abstract

Dietary fibres are frequently used for the treatment of obesity. The aim of this study was to evaluate the efficacy of *Irvingia gabonensis *seeds in the management of obesity. This was carried out as a double blind randomised study involving 40 subjects (mean age 42.4 years). Twenty-eight subjects received *Irvingia gabonensis *(IG) (1.05 g three time a day for one month) while 12 were on placebo (P) and the same schedule. During the one-month study period all subjects were on a normocaloric diet evaluated every week by a dietetic record book. At the end, the mean body weight of the IG group was decreased by 5.26 ± 2.37% (p < 0.0001) and that of the placebo group by 1.32 ± 0.41% (p < 0.02). The difference observed between the IG and the placebo groups was significant (p < 0.01). The obese patients under *Irvingia gabonensis *treatment also had a significant decrease of total cholesterol, LDL-cholesterol, triglycerides, and an increase of HDL-cholesterol. On the other hand, the placebo group did not manifest any changes in blood lipid components.* Irvingia gabonensis *seed may find application in weight lose.

## Introduction

Obesity is of major primary care concern and is targeted as an international health objective in Healthy 2000, which seeks to reduce the prevalence of obesity to less than 20%. In the last 10 years, the number of overweight people has increased from 26 to 34% [1]. Conventional dietary and behavioral treatment have failed in long-term management. Dietary strategies used to manage obesity include the use of high fibre, low carbohydrates and fats diet [2,3]. Beneficial effect of dietary fibre in the management of obesity is not well established, since their mechanism of action is not known. The discovery of new medicinal plants has led to the creation of potential drugs that modify feeding behavior and metabolism and may therefore have application in weight management. The aim of the present study is to investigate the effect of *Irvingia gabonensis *extract on body weight.

## Subjects and methods

A total of 40 obese subjects aged between 19 and 55 years were selected from a group responding to a radio advertisement. After physical examination and laboratory screening tests, diabetics, pregnant and lactating women were excluded. None of these subjects took any weight reducing medication and none was following any specific diet. The purpose, nature and potential risks of the study were explained to all patients and a written informed consent was obtained before their participation. The local research ethics committee approved the experimental protocol.

### Study design

The study was as a randomised, double blind placebo-controlled crossover design, and consisted of a 4-week treatment period. Subjects were given two different types of capsules containing 350 mg of *Irvingia gabonensis *seed extract (active formulation) or oat bran (placebo). Three capsules were taken three times daily, one-half hour before meals (a total daily amount of 3.15 g of *Irvingia gabonensis *seed extract) with a glass of warm water. Capsules were identical in shape, colour and appearance, with neither patients nor researchers knowing what capsule they received. During the experimental period, subjects were examined weekly, with their body weight, body fat, waist and hip circumferences recorded each time. Subjective findings such as increased or decreased appetite, feeling of lightness and gastrointestinal pains were individually noted. Side effects of the active extract, if any were also solicited and noted during each visit. The subjects were also interviewed about their physical activity and food intake during the trial, and were instructed to eat a low fat diet (1800 Kcal) as well as keep a record for seven consecutive days (using household measurements).

### Anthropometric measurements

Anthropometric measurements were done at each visit, with body weight and body fat (impedance measurement using a TANITA™ monitor Scale) measurements on fasted (12 hour) subjects wearing light clothing. Waist and hip circumferences were measured by soft non-stretchable plastic tape on the narrowest and the widest parts of the trunk.

### Laboratory methods

Blood samples were collected after a 12 h overnight fast into heparinized tubes at the beginning of the study, after two weeks and at the end (4 weeks) of treatment. The concentrations of total cholesterol, triacylglycerol, HDL-cholesterol, and glucose, in plasma were measured using a commercial diagnostic kit (Cholesterol infinity, triglycerides Int, EZ HDL™ cholesterol, EZ LDL™ cholesterol, Glucose Trinder, respectively) from SIGMA Diagnostics

### Statistical Analysis

Results are expressed as mean ± SEM except for anthropometric measurements. Paired Student's t-test was carried out on the start and end values of placebo and *Irvingia gabonensis *capsules and also on the differences between the placebo and *Irvingia gabonensis *crude extract.

## Results

### Effect on body composition

*Irvingia gabonensis *induced a decrease in weight of 2.91 ± 1.48% (p < 0.0001) after two weeks and 5.6 ± 2.7% (p < 0,0001) after one month. Although the percentage of body fat was not significantly reduced with both placebo and IG, the waist circumference (5.07 ± 3.18%; p < 0.0001) and hip circumference (3.42 ± 2.12%; p < 0,0001) were significantly reduced by IG. A reduction of 1.32 ± 0.41% (p < 0.02) and 2.23 ± 1.05% (p < 0.05) was observed with the placebo after two and four weeks respectively of treatment.

### Effect on blood pressure

From the second week of experimentation, the systolic blood pressure was significantly reduced by the active extract (Table 2).

**Table 2:** Effect Irvingia gabonensis on systolic (SBP) and diastolic (DBP) blood pressure

	Treatment period (weeks)			
	
		0	2	4
SBP (mmHg)	Active	136.41 ± 19.57	132.66 ± 18.48*	132.83 ± 17.97*
	Placebo	134 ± 5.05	121.5 ± 5.89	123.83 ± 2.92
DBP (mmHg)	Active	98.5 ± 19.52	97.5 ± 22.80	94.08 ± 11.07
	placebo	93.50 ± 10.31	93.83 ± 7.41	91.5 ± 6.53

Values are means ± sem. Significant differences were at *P < 0.001 by comparison to the placebo group.

### Effect on blood lipids components

The plasma total cholesterol cencentration was reduced by 39.21%, triglyerides by 44.9% (p < 0,05) and LDL by 45.58%. This was accompanied by a significant increase in HDL-cholesterol of 46.852%. The CT/HDL ratio (p < 0.05) and the blood glucose level were also reduced (32.36%; p < 0.05). No significant change was observed in the placebo group.

## Discussion

The soluble fibre of the seed of *Irvingia gabonensis *like other forms of water-soluble dietary fibres, are "bulk-forming" laxatives. *Irvingia gabonensis *seeds delay stomach emptying, leading to a more gradual absorption of dietary sugar. This effect can reduce the elevation of blood sugar levels that is typical after a meal [4]. Controlled studies have found that after-meal blood sugar levels are lower in people with diabetes given glucomannan in their food [5] and overall diabetic control is improved with soluble fibre-enriched diets according to preliminary [6] and controlled [7,8] trials. One double-blind study reported that glucomannan (8–13 grams per day) stabilized blood sugar levels in people with the insulin resistance syndrome [9]. Like other soluble fibers, *Irvingia gabonensis *seed fibre can bind to bile acids in the gut and carry them out of the body in the faeces, which requires the body to convert more cholesterol into bile acids [10]. This can result in the lowering of blood cholesterol as well as other blood lipids. Controlled double-blind [11,12] studies have shown that supplementation with several grams per day of soluble fibre significantly reduced total blood cholesterol, LDL cholesterol, and triglycerides and in some cases raised HDL cholesterol, these being comparable with effects noticed with *Irvingia gabonensis.*

Considering the wide use of *Irvingia gabonensis *in the preparation of various dishes in Cameroon, its use should be further encouraged for the purposes of control of dietary lipids as well as for weight reduction.

**Table 1:** Effect of *Irvingia gabonensis *crude extract on body weight, body fat, waist and hip circumferences

	Treatment period (weeks)			
	
		0	2	4
Weight (kg	Active	105.10 ± 16.98	102.3 ± 17.06	101.01 ± 16.63
	Placebo	79.43 ± 9.83	79.43 ± 9.83	79.33 ± 10.63
	Active	46.11 ± 4.4848	46.5 ± 3.68	45.34 ± 3.52
Body fat (%)	Placebo	40.58 ± 3.49	40.58 ± 3.9	40.3 ± 3.8
	Active	112.76 ± 20.5	109.7 ± 20.4	106.6 ± 20.79
Waist (cm)	Placebo	81.1 ± 7.1	81,91 ± 7,91	81.25 ± 7.52
	Active	125.69 ± 11.34	122.92 ± 10.67	121.15 ± 10.39
Hip (cm)	Placebo	122.2 ± 10.7	122.2 ± 10.7	121.5 ± 10.9

**Table 3:** The effect of Irvingia gabonensis on blood total cholesterol (TC), triglyceride (TRI), high density lipoprotein cholesterol (HDL-c), low density lipoprotein cholesterol (LDL-c) and glucose.

		T-cholesterol	TRI	HDL-c	LDL-c	LDL/HDL	T-cho/HDL	GLUCOSE
Active	Initial	215 ± 55.12	162 ± 33.15	61.23 ± 20.36	121.37 ± 36.3	1.98 ± 1.78	3.51 ± 2.70	3.8 ± 1.92
	Final	130.68 ± 39.5	89.22 ± 55.63	89.9 ± 28.44	66.08 ± 34.27	0.735 ± 1.20	1.45 ± 1.38	2.57 ± 1.03
Placebo	Initial	163.70 ± 25.32	130.65 ± 37.82	31.38 ± 25.21	105.06 ± 11.86	5.05 ± 3.94	6.44 ± 3.37	3.6 ± 0.41
	Final	158.36 ± 30.46	100.52 ± 32.55	41.20 ± 19.53	98.55 ± 27.99	3.19 ± 1.85	4.51 ± 2.07	3.9 ± 0.74
